# Comparative bioavailability of three benznidazole formulations in healthy individuals: a randomised study

**DOI:** 10.1590/0074-02760250307

**Published:** 2026-07-10

**Authors:** Gabriel Parreiras Estolano da Silveira, Laís Bastos da Fonseca, Rita Estrela, Douglas Pereira Pinto, João Fellipe Garcia Medeiros de Araújo, Luiz Villarinho Pereira Mendes, Eloan Pinheiro, Fernanda de Souza Nogueira Sardinha Mendes, Debbie Vermeij, Andréa Silvestre de Sousa

**Affiliations:** 1 Fundação Oswaldo Cruz-Fiocruz, Serviço de Equivalência e Farmacocinética, Rio de Janeiro, RJ, Brasil; 2 Fundação Oswaldo Cruz-Fiocruz, Escola Nacional de Saúde Pública Sergio Arouca, Rio de Janeiro, RJ, Brasil; 3 Fundação Oswaldo Cruz-Fiocruz, Instituto Nacional de Infectologia Evandro Chagas, Rio de Janeiro, RJ, Brasil; 4 Universidade Federal do Rio de Janeiro, Faculdade de Farmácia, Laboratório de Farmacometria, Rio de Janeiro, RJ, Brasil; 5 Fundação Oswaldo Cruz-Fiocruz, Rio de Janeiro, RJ, Brasil; 6 Universidade Federal do Rio de Janeiro, Faculdade de Medicina, Departamento de Medicina Interna, Rio de Janeiro, RJ, Brasil

**Keywords:** benznidazole, bioequivalence, bioavailability

## Abstract

**BACKGROUND:**

Benznidazole (BZN) has been used for more than fifty years in the treatment of Chagas disease (CD). It is produced by only three pharmaceutical companies worldwide: Lafepe (Brazil); Elea (Argentina) and Liconsa (Spain). The therapeutic interchangeability among these products has never been evaluated.

**OBJECTIVES:**

To assess bioequivalence between three 100 mg BZN formulations.

**METHODS:**

Pharmaceutical equivalence, with dissolution testing, was assessed prior to the bioequivalence study. For bioequivalence, healthy adult participants received 100 mg of BZN formulations after meal, in a randomised clinical trial. Blood BZN concentrations were measured. Pharmacokinetic parameters were determined by non-compartmental analysis.

**FINDINGS:**

The BZN Liconsa demonstrated faster *in vitro* dissolution. However, bioavailability was not different between formulations. The mean area under the curve (AUC)84h values were 49431.22 h*ng/mL (SD = 9938.53) to Lafepe, 48974.38 h*ng/mL (SD = 10304.67) to Elea and 48204.17 h*ng/mL (SD = 9342.18) to Liconsa. The mean Cmax values were 2339.23 ng/mL (SD = 445.53) for Lafepe, 2209.04 ng/mL (SD = 448.02) for Elea and 2303.90 ng/mL (SD = 431.41) for Liconsa. The mean tmax was not different between formulations. AUC were higher in women for Elea (20%) and Lafepe (27%). The adverse events did not differ between sexes.

**MAIN CONCLUSIONS:**

The 100 mg BZN formulations demonstrated bioequivalence.

Discovered in 1909 by Brazilian scientist Carlos Chagas, Chagas disease (CD) is a neglected tropical disease caused by the flagellated protozoan *Trypanosoma cruzi*. It is endemic in 21 Latin American countries and carries significant health and socioeconomic burdens. Although previously confined to vulnerable, mainly rural endemic areas, CD has progressively spread beyond the Americas, with cases now reported on five continents.^1^ ^2^ An estimated 8 million people worldwide are infected, and approximately 100 million people are at risk of infection. However, these numbers are likely underestimated, as less than 10% of infected individuals are diagnosed, and approximately 1% has received etiological treatment.^1^ ^3^

There is currently no vaccine for CD, and treatment still relies on two medications that were licensed over 50 years ago: nifurtimox (NFX), launched by Bayer in 1965; and benznidazole (BZN), introduced by Roche in 1971.^4^ ^5^ Comparative data on BZN and NFX remain inconclusive in terms of efficacy. Both BZN and NFX are recommended as first-line therapies for CD by Pan American Health Organization (PAHO) / World Health Organisation (WHO) guidelines and may be used interchangeably in terms of therapeutic efficacy.^6^ However, BZN is more frequently used due to its simpler dosing regimen and potentially better tolerability regarding adverse events.^7^ ^8^

BZN (N-benzyl-2-nitro-1-imidazole acetamide) is a 2-nitroimidazole derivative with antiprotozoal activity. It is active against multiple life-cycle stages of *T. cruzi*, including intracellular amastigotes, although susceptibility may vary among parasite genotypes and dormant, non-replicative forms may show reduced sensitivity. In the acute phase of CD or during its reactivation, BZN demonstrates an effectiveness rate of 65-80%, with a significant chance of cure. Some studies also suggest efficacy in chronic phase, except in advanced chronic Chagas heart disease, where BZN significantly reduced parasitaemia, but it did not translate into improvement in the evaluated clinical outcomes.^9^ ^10^ ^11^ ^12^ ^13^

Understanding the pharmacokinetic (PK) profile of BZN is essential for optimising treatment regimens in patients with CD. For over four decades, PK data were based on formulations that are no longer available, such as Rochagan® and Radanil®, previously produced by Hoffmann-La Roche. In 2003, BZN production was transferred to the Laboratório Farmacêutico do Estado de Pernambuco Governador Miguel Arraes (Lafepe), a government-affiliated Brazilian manufacturer, initially using the active pharmaceutical ingredient (API) donated by Roche. Since 2011, Lafepe has sourced its API from Nortec Química (Brazil).^14^

In Argentina, production began in 2012 through the private Laboratorio Elea Phoenix SA (Abarax®), in collaboration with the Ministry of Health and the Non-Governmental Organisation Fundación Mundo Sano, using API from Maprimed Farmoquimico Laboratory (Argentina). By 2014, Elea partnered with Liconsa SA (Chemo Group, Spain), resulting in a formulation that received Food and Drug Administration (FDA) approval in 2017 for paediatric use (ages 2-12 years). The FDA registration identifies Empresa Quimica Sintética (Spain), also part of the Chemo Group, as the API supplier for Liconsa. Currently, three manufacturers produce BZN, each relying on different API suppliers.^14^

Liconsa has been registered with the FDA as a reference standard for the 100 mg BZN dose since August 2017. Lafepe obtained reference product status in Brazilian market in 2024 (one year after this study), and Elea is authorised in Argentina. These three products occupy a unique position in their respective markets, with no registered generic or similar alternatives.^14^ However, the lack of established bioequivalence among them limits therapeutic interchangeability, restricts patient access to therapy, and difficulties the interpretation and generalisation of clinical study results involving these formulations.

More recently, pharmacokinetic studies using currently marketed formulations from Lafepe and Elea have emerged,^15^ ^16^ ^17^ although without explicit bioequivalence assessments. The present study provides novel data by directly comparing the pharmacokinetic profiles and bioequivalence of three currently available 100 mg BZN formulations (Lafepe, Elea, and Liconsa) under controlled conditions in healthy subjects.

This study aimed to compare the bioavailability of 100 mg BZN tablets manufactured by Lafepe, Elea, and Liconsa following a single oral dose administrated to healthy individuals after a standardised meal. Additionally, the influence of sex on pharmacokinetic parameters and bioequivalence were evaluated.

## MATERIALS AND METHODS

*Pharmaceutical equivalence* - Prior to the bioequivalence study, pharmaceutical equivalence was assessed through in vitro tests, including physicochemical and dissolution evaluations. Three drug formulations were analysed: Benznidazole Lafepe, manufactured by Lafepe (batch 21030012); Abarax, manufactured by Elea Phoenix SA (batch 3218); and Benznidazole Chemo, manufactured by Liconsa SA (batch LC60657).

Every pharmaceutical equivalence study includes quality control tests and a dissolution profile to ensure drug compliance before proceeding to bioequivalence testing. In this study, assessments were conducted based on the monograph of Benznidazole Lafepe. It is important to note that no monograph exists in the pharmacopoeias recommended by the Brazilian Health Regulatory Agency (Anvisa).

The tests performed included evaluation of appearance, identification, average weight, individual weight variation, friability, hardness, disintegration, assay, content uniformity, related substances, dissolution, and, finally, the comparative dissolution profile.

For the dissolution test, a Distek dissolution tester system (Model 6100 Evolution) equipped with USP Apparatus 2 was used. The test was performed in 1000 mL of 0.1 M hydrochloric acid (HCl) at 37ºC, using the paddle method at a stirring rate of 100 rpm. Samples were collected at 10, 15, 30, 60, 90, 120, and 240 min. For analysis, samples were diluted 1:5 in the dissolution medium. The extend of drug dissolution was assessed using a UV-VIS spectrophotometer (Mettler Toledo, Model UV 5) at an optimal wavelength of 324 nm. These physicochemical tests adhered to the parameters specified in Brazilian regulatory guidelines.^18^

*Bioequivalence study* - The *in vivo* study was a single-centre, open-label, randomised crossover trial using three drug formulations of 100 mg BZN tablets: Lafepe, designated as the test formulation (T); Elea, designated as comparator formulation one (R1); and Liconsa, designated as comparator formulation two (R2). The same batches evaluated in the pharmaceutical equivalence test were used. Treatments were administered as a single 100 mg dose in three periods, according to a randomised sequences of treatment (R1TR2, R2R1T, TR2R1), following a Latin Square design. The randomisation list was generated using the PROC PLAN procedure in SAS® software version 9.4. A seven-day washout period was implemented between treatments.

The bioanalytical analyses were conducted in a laboratory certified by Anvisa, in compliance with Brazilian regulatory requirements for bioanalytical studies.^19^ All analytical equipment used in the study undergoes routine qualification, calibration, and preventive maintenance in accordance with the laboratory's quality management system and applicable regulatory standards.

*Study population, blood samples and adverse events monitoring* - Healthy male and female participants aged 18 to 50 years were recruited from a single clinical research centre to ensure standardised study conditions and minimise operational variability. Baseline laboratory tests [renal and hepatic function, lipid profile, complete blood count, and viral markers for hepatitis B and C and anti-human immunodeficiency virus (HIV)] were performed to confirm participant eligibility (healthy condition). Exclusion criteria included liver or gastrointestinal diseases that could affect BZN absorption or metabolism, pregnancy, breastfeeding, smoking, and a history of alcohol or drug abuse. Use of any other medications was prohibited for two weeks before the study, except for dipyrone, paracetamol, and contraceptives. Participants were also required to abstain from alcohol for 48 h before the study, and were instructed to follow standard dietary restrictions, including overnight fasting prior to drug administration, as commonly applied in pharmacokinetic studies.

Participants underwent three confinement periods of 36 h each, with a seven-day interval between them. In each period, BZN tablets were administered together 200 mL of water, 30 min after a standardised high-fat, high-calorie meal (872.4 calories, with 53.0% fat), which was consumed following a minimum fasting period of 10 h, in accordance with regulatory recommendations for bioequivalence studies conducted under fed conditions to evaluate potential food effects on drug absorption.^20^ ^21^

Blood samples were collected from each participant 30 min before BZN administration (time 0.0 h, baseline), and at 25 time points post-dose: 0.5; 1.0; 1.5; 2.0; 2.5; 3.0; 3.5; 4.0; 4.5; 5.0; 5.5; 6.0; 6.5; 7.0; 7.5; 8.0; 10.0; 12.0; 14.0; 24.0; 36.0; 48.0; 60.0; 72.0; and 84.0 h. Blood samples were collected in tubes containing EDTA K3 anticoagulant and centrifuged at 3000 rpm for 10 min at 4ºC (± 2ºC) to separate the plasma fraction, which was immediately stored in an ultra-freezer at -70ºC (± 15ºC) until analysis.

Following each BZN administration, participants were clinically observed for 24 h at the clinical research unit, with monitoring performed by trained medical staff, including vital signs assessment, protocol-specified laboratory evaluations, and monitoring for adverse reactions.

*Determination of BZN in blood plasma* - Regulatory agency (Anvisa) recommend the analysis of unchanged BZN in bioequivalence studies.^20^ Accordingly, BZN was extracted from plasma using a liquid-liquid extraction method with methyl tert-butyl ether (MTBE). For each 100.00 μL of plasma, 50.00 μL of an internal standard working solution [metronidazole (MTZ), 1.00 µg/mL] and 1000.00 μL of MTBE were added. The samples were mixed using an automatic shaker for 1 min and centrifuged for 5 min at 14400 rpm and 10ºC (± 2ºC). Subsequently, 600.00 μL of the supernatant (organic phase) was transferred to a polypropylene microtube, evaporated under a stream of nitrogen gas, and reconstituted with 300.00 μL of dilution solution composed of methanol:acetonitrile (80:20) with 0.10% formic acid in Type I water (70:30 v/v). The samples were mixed at 1500 rpm for 1 minute, and 300.00 μL aliquots were transferred to analysis vials.

BZN quantification was performed using mass spectrometry (API 4000™ AB SCIEX) in positive electrospray ionisation mode (ESI+), combining high-performance liquid chromatography (HPLC) with tandem mass spectrometry (MS/MS). The transitions monitored were 261.092 → 91.100 m/z for BZN and 172.099 → 127.975 m/z for MTZ. The BZN calibration curve was linear from 25.00 to 4000.00 ng/mL, with MTZ internal standard maintained at a fixed concentration of 500.00 ng/mL. An ACE 5 μm C18 (150 mm x 4.6 mm) analytical column was used for chromatographic separation.

The analytical method was developed and validated in accordance with Brazilian regulatory guideline.^22^

*Pharmacokinetic parameters* - Pharmacokinetic parameters were determined by non-compartmental analysis (NCA). The maximum plasma concentration (Cmax) and time to reach Cmax (tmax) were obtained directly from the individual plasma concentration-time curve. The area under the plasma concentration-time curve from time zero to the last measurable concentration within 84 h [area under the curve (AUC)84h] was calculated using the linear trapezoidal method. The total AUC from time zero to infinity (AUC∞) was calculated as AUCt + (Ct/λ), where Ct is the last quantifiable concentration and λ is the apparent terminal elimination rate constant. The plasma elimination half-life (t1/2) was calculated as ln(2)/λ. Apparent total body clearance (CL) and the apparent volume of distribution in the terminal phase (Vz) were calculated using the equations CL = Dose/AUC∞ and Vz = Dose/(λ*AUC∞), respectively, expressed in relation to the bioavailability factor (F) as CL/F and Vz/F.

Pharmacokinetic parameters were determined for each participant and formulation. To evaluate sex-related differences, the nominal administered dose (100 mg) was normalised by each participant's body weight. The weight was measured at each study period. The resulting weight-adjusted dose (mg/kg) was used to adjust individual pharmacokinetic parameters. All analyses were conducted using Phoenix WinNonlin® version 8.4 (Certara).

*Statistical analysis* - Mean plasma concentration versus time curve were plotted to visually compare the three formulations. Descriptive statistics were calculated for each pharmacokinetic parameters and formulation, for bioequivalence test.

Bioequivalence was assessed using average bioequivalence criteria. A linear mixed-effects model was applied to the log-transformed AUC and Cmax values, including sequence, period and formulation as fixed effects, and subject nested within the sequence as a random effect. Bioequivalence was concluded if the 90% confidence intervals (90% CI) for the geometric mean ratios (T/R1, T/R2, and R1/R2) of the PK parameters AUC and Cmax were within the acceptance range of 80% to 125% for 90% CI, following regulatory guidance.^20^ ^23^ ^24^

Comparisons between sex and formulations were performed, using the Mann-Whitney Rank Sum test. Differences were considered statistically significant at p < 0.05.

Statistical analyses were conducted using validated software packages, including SAS® 9.4 (SAS Institute Inc.), Phoenix WinNonlin® 8.4 (Certara), and SigmaPlot® 14.1 (Systat Software).

*Ethical aspects* - Pharmaceutical equivalence and bioequivalence analyses were performed in research centres certified by Anvisa. The study was conducted in accordance with Resolution No. 466/2012 of the Brazilian National Health Council^25^ and approved by the Institutional Research Ethics Committee (CAAE: 63382222.4.0000.8098; approval document No. 5.670.824; approved on September 28, 2022). The study protocol was registered in the Brazilian Registry of Clinical Trials (ReBEC) under ID: 63382222.4.0000.80981 and is available at: https://ensaiosclinicos.gov.br/rg/RBR-6zfzs4j.

## RESULTS

**Fig. 1:**
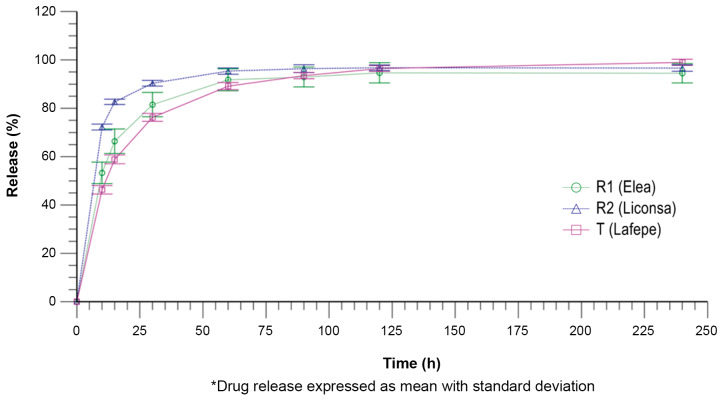
dissolution profiles of the three benznidazole (BZN) 100 mg formulations obtained during the pharmaceutical equivalence test.

*Pharmaceutical equivalence tests* - The drug formulations complied with regulatory guidance for all physicochemical tests (Table I). However, the dissolution profile analysis revealed that Lafepe and Liconsa formulations were not pharmaceutically equivalent, with an F₂ value of 42. The Liconsa formulation exhibited a faster dissolution rate, and it was only after 90 min that both formulations showed similar percentages of drug dissolved (Table II). In contrast, the comparison between Elea and Lafepe yielded an F₂ value of 65, meeting the regulatory threshold for pharmaceutical equivalence (Fig. 1).

**TABLE I:** Results of pharmaceutical equivalence

Tests	Elea (R1)	Liconsa (R2)	Lafepe (T)
Appearance	White, circular, flat tablet with a bisecting score on one side	White, circular, flat tablet, bisecting scores on both sides and the letter "E" inscribed in each quadrant on one side	White, circular, flat tablet with a bisecting score on one side
Identification	The sample solution of the test drug exhibited absorbance at a wavelength of 324 nm	The sample solution of the test drug exhibited absorbance at a wavelength of 324 nm	The sample solution of the test drug exhibited absorbance at a wavelength of 324 nm
Average weight	299.61 mg	304.26 mg	252.98 mg
Friability	0.04%	0%	0.26%
Hardness	48 N	62 N	93 N
Disintegration	Last tablet: 30s	Last tablet: 2 min and 32 s	Last tablet: 1 min and 03 s
Assay	102.9%	102.1%	104.6%
Content uniformity	VA = 5.7	VA = 3.6	VA = 3.9
Related substances*	Not detected	Not detected	Not detected
Dissolution	Stage E1 60 min: minimum of 90.9%; 120 min: minimum of 95.3%	Stage E1 60 min: minimum of 88.4%; 120 min: minimum of 90.0%	Stage E1 60 min: minimum of 85.3%; 120 min: minimum of 93.2%

* 2-Aminoimidazole sulfate; 2-Nitroimidazole (impurity A); N-Benzylchloroacetamide (impurity B).

**TABLE II:** Dissolution test for 100 mg benznidazole (BZN) tablets from Elea (R1), Liconsa (R2), and Lafepe (T)

Time (min)	Elea (R1)		Liconsa (R2)		Lafepe (T)	
	Q	RSD	Q	RSD	Q	RSD
0	0.00	0.00	0.00	0.00	0.00	0.00
10	53.30	8.34	72.26	1.68	46.32	3.79
15	66.37	7.65	82.65	1.31	58.85	3.15
30	81.52	6.16	90.38	1.27	76.24	2.19
60	91.81	4.94	95.42	1.36	89.18	1.64
90	92.95	4.43	96.41	1.68	93.53	1.40
120	94.67	4.43	96.79	1.14	96.46	1.26
240	94.52	4.24	96.65	1.42	99.01	1.31

Values are described in percentual (%). Q: dose/solubility ratio (% mean); RSD: residual standard deviation (%).

**Fig. 2:**
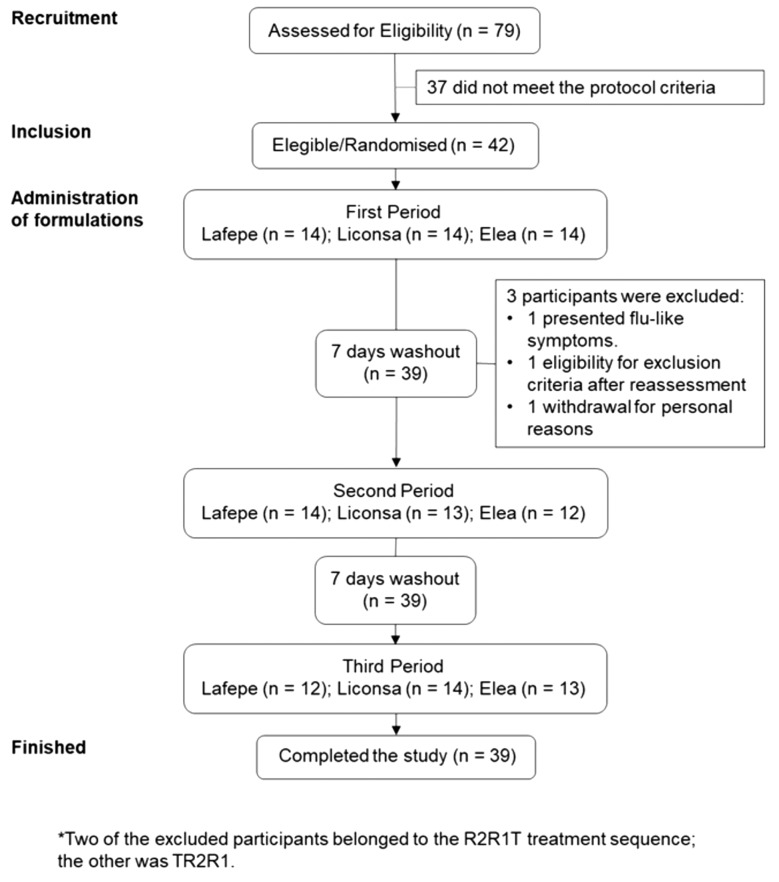
flowchart to bioequivalence analysis.

**Fig. 3:**
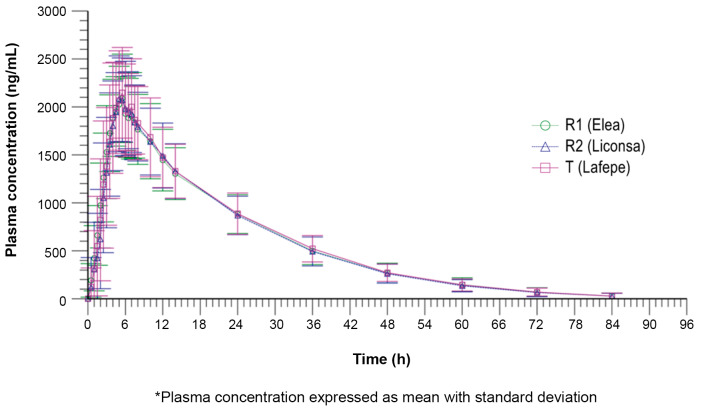
plasma concentration versus time for 100 mg benznidazole (BZN) tablets from Elea (R1), Liconsa (R2), and Lafepe (T).

*Bioequivalence analysis* - A total of 42 healthy participants residing in Campinas, São Paulo, Brazil, were enrolled in the bioequivalence study, in 2022. Of these, 39 participants completed the study, 17 men and 22 women, as demonstrated in Fig. 2. The overall median was 36 years (IQR 30-42) [37.50 years for women (IQR 30.75-41.25); 36 years for men (IQR 27-42)] and weight median was 69.1 kg (IQR 64.30-83.10) [66.80 kg for women (IQR 61.88-70.65); 83.10 kg for men (IQR 67.30-89.20)]. The mean plasma concentration versus time curve for each drug formulation is presented in Fig. 3, and the average pharmacokinetic parameters are summarised in Table III.

Bioequivalence was assessed by comparing the bioavailability parameters (Cmax and AUC) among the three drug formulations. The 90% CI for the ratios of geometric means (AUC and Cmax for T/R1, T/R2, R2/R1) fell within the accepted bioequivalence range of 0.80 to 1.25 (Table IV). Differences in Cmax and AUC between the drug formulations were minimal, not exceeding 6% and 3%, respectively. Intraindividual variability for the pharmacokinetic parameters was low, under 10% for Cmax and under 7% for AUC. Statistical power exceeded 99,99% for all comparations. The tmax was approximately 5 h for all formulations, not differing statistically between formulations.

**Fig. 4:**
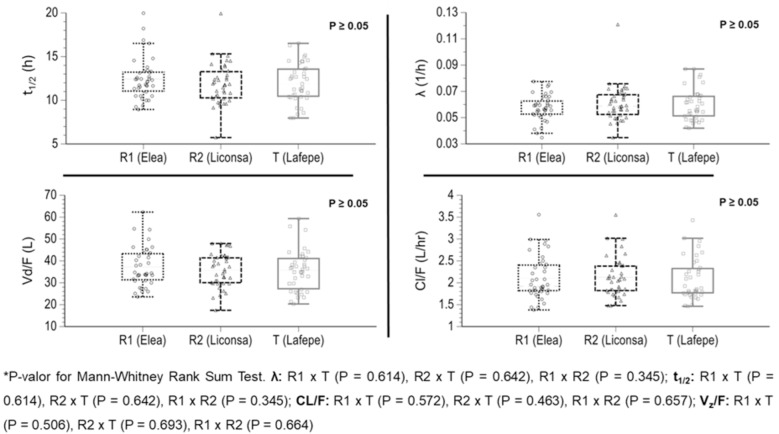
box plot to distribution and elimination pharmacokinetic parameters by drug formulation.

Distribution (Vz/F) and elimination parameters (CL/F, λ, and t1/2) also showed no significant differences among the three formulations, as illustrated in Fig. 4.

**TABLE III:** Pharmacokinetics parameters for 100 mg benznidazole (BZN) tablets from Elea (R1), Liconsa (R2), and Lafepe (T)

Variable	Units	N	R1 (Elea)	R2 (Liconsa)	T (Lafepe)
tmax	(h)	39	4.95 (1.22)	4.71 (1.20)	4.94 (1.42)
Cmax	(ng/mL)	39	2209.04 (448.02)	2303.9 (431.41)	2339.23 (445.53)
AUC84h	(h*ng/mL)	39	48974.38 (10304.67)	48204.17 (9342.18)	49431.22 (9938.53)
AUC∞	(h*ng/mL)	39	49910.49 (10608.09)	48992.55 (9590.13)	50292.7 (10128.11)
%AUCextrap	(%)	39	1.85 (0.91)	1.59 (0.79)	1.72 (0.82)
λ	(h^-1^)	39	0.0574 (0.0096)	0.0609 (0.0136)	0.0600 (0.012)
t1/2	(h)	39	12.45 (2.35)	11.86 (2.36)	11.97 (2.26)
CL/F	(L/h)	39	2.1 (0.49)	2.13 (0.46)	2.08 (0.47)
Vz/F	(L)	39	37.23 (8.91)	35.8 (7.65)	35.61 (9.21)

Values are described as mean (standard deviation). tmax: time to reach Cmax; Cmax: maximum concentration; AUC84h: area under the curve up to the last measurable concentration within 84 h; AUC∞: area under the curve up to infinity; %AUCextrap: extrapolated percentage of AUC∞ in relation to AUC84h; λ: apparent elimination constant; t1/2: elimination half-life; CL/F: apparent total body clearance; Vz/F: apparent volume of distribution.

**TABLE IV:** Bioequivalence analysis for 100 mg benznidazole (BZN) tablets from Elea (R1), Liconsa (R2) and Lafepe (T)

**T (Lafepe) X R1 (Elea)**					
**Parameter**	**N**	Ratio T/R1*	CI 90%**	**Power**	CVintra (%)***
Ln (AUC84h)	39	100.86	(98.43; 103.34)	> 99.99	6.32
Ln (AUC∞)	39	100.69	(98.26; 103.18)	> 99.99	6.33
Ln (Cmax)	39	106.72	(102.90; 110.67)	> 99.99	9.44
tmax (diference)	39	0.00	(-0.50; 0.50)	-	
**T (Lafepe) X R2 (Liconsa)**					
**Parameter**	**N**	Ratio T/R2*	CI 90%**	**Power**	CVintra (%)***
Ln (AUC84h)	39	102.35	(99.89; 104.88)	> 99.99	6.32
Ln (AUC∞)	39	102.47	(100.00; 105.01)	> 99.99	6.33
Ln (Cmax)	39	101.90	(98.26; 105.68)	> 99.99	9.44
tmax (diference)	39	0.00	(0.00; 0.50)	-	
**R2 (Chemo) X R1 (Elea)**					
**Parameter**	**N**	Ratio R2/R1*	90% CI**	**Power**	CVintra (%)***
Ln (AUC84h)	39	98.54	(96.17; 100.97)	> 99.99	6.32
Ln (AUC∞)	39	98.26	(95.89; 100.69)	> 99.99	6.33
Ln (Cmax)	39	104.72	(100.98; 108.61)	> 99.99	9.44
tmax (diference)	39	0.00	(-0.50; 0.00)	-	

* Geometric mean ratio;

** Confidence interval of 90%;

*** Intra-subject variability (CVintra).

**Fig. 5:**
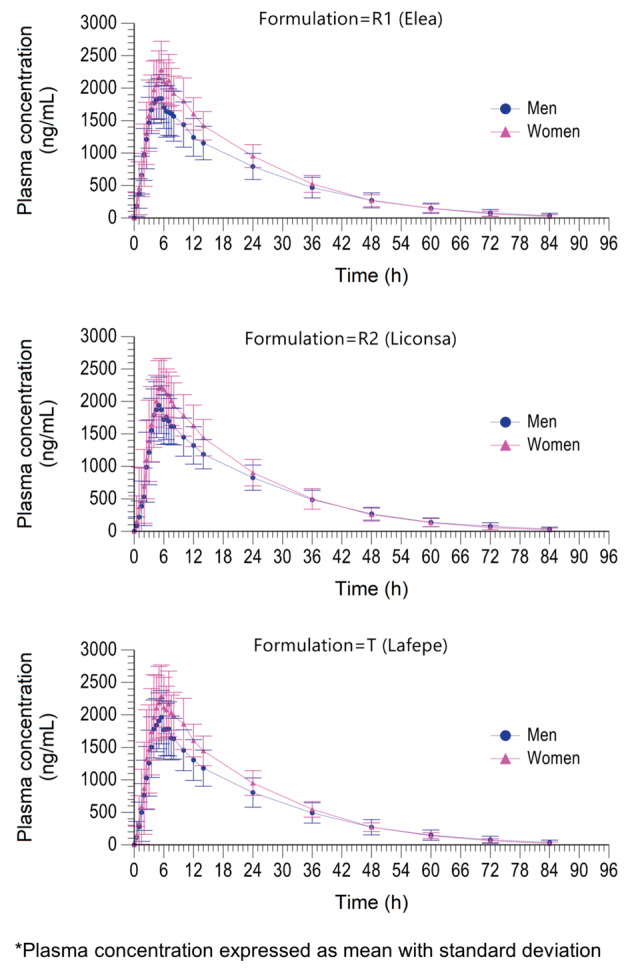
plasma concentration versus time for Elea (R1), Liconsa (R2) and Lafepe (T) by sex.

*Sex-based differences* - Plasma concentrations were higher in women for all three formulations (Fig. 5). AUC was higher in women for Elea and Lafepe formulations and Cmax was higher in women for all (Table V). Female participants had a lower median body weight than male participants (20%), resulting in a more pronounced weight-adjusted dose (mg/kg) in women (25%). Consequently, women could exhibit higher Cmax and AUC values. After normalising pharmacokinetic parameters by weight (AUC/D and Cmax/D), no statistically significant differences between sexes were observed. Analysis of covariance (ANCOVA) confirmed that body weight was a significant covariate in the comparison between sexes (p < 0.001), influencing BZN exposure. There was no significant interaction between sex and weight for the AUC.

**TABLE V:** Pharmacokinetic parameters by drug formulation and sex

Pharmacokinetic parameter	Formulation	Groups		P**
		Women (N = 22)*	Men (N = 17)*	
Cmax (ng/mL)	R1 (Elea)	2407.95 [2154.30; 2616.70]	1844.93 [1635.53; 226.14]	< 0.001***
	R2 (Liconsa)	2306.68 [2091.97; 2752.80]	2093.87 [1924.29; 2267.72]	0.016***
	T (Lafepe)	2518.08 [2193.13; 2731.85]	1949.65 [1807.45; 2495.45]	0.002***
Cmax/D (kg*ng/mL/mg)	R1 (Elea)	1610.71 [1472.60; 1714.71]	1537.63 [1447.77; 1627.51]	0.234
	R2 (Liconsa)	1633.19 [1507.24; 1704.81]	1674.98 [1521.06; 1838.65]	0.322
	T (Lafepe)	1648.19 [1511.68; 1802.63]	1613.87 [1551.67; 1728.95]	0.910
tmax (h)	R1 (Elea)	5.50 [4.50; 6.13]	4.50 [3.75; 5.00]	0.007***
	R2 (Liconsa)	5.00 [4.50; 5.50]	4.50 [4.00; 5.00]	0.032***
	T (Lafepe)	5.25 [4.00; 5.50]	4.50 [4.25; 5.50]	0.451
AUC84h (h*ng/mL)	R1 (Elea)	52717.19 [49610.63; 55940.26]	43690.48 [35214.86; 53407.54]	0.023***
	R2 (Liconsa)	52144.60 [45627.48; 55623.98]	45835.82 [37258.63; 50862.47]	0.084
	T (Lafepe)	54324.24 [45127.79; 57207.84]	42785.41 [35974.71; 55130.04]	0.027***
AUC84h/D (kg*h*ng/mL/mg)	R1 (Elea)	35280.60 [31410.69; 37949.33]	33541.27 [29662.07; 39053.13]	0.821
	R2 (Liconsa)	33927.06 [30118.84; 37360.88]	37221.94 [30383.15; 39650.55]	0.365
	T (Lafepe)	36232.02 [32021.38; 39157.72]	34493.03 [30192.09; 38171.90]	0.734
AUC∞ (h*ng/mL)	R1 (Elea)	53332.46 [50420.20; 56905.19]	44300.73 [35842.26; 54503.23]	0.025***
	R2 (Liconsa)	52718.79 [46374.63; 56336.82]	46421.73 [37857.33; 51914.27]	0.095
	T (Lafepe)	55368.61 [45685.86; 57991.85]	43423.41 [36621.87; 56114.66]	0.041***
AUC∞/D (kg*h*ng/mL/mg)	R1 (Elea)	35737.87 [37769.48; 38324.88]	34160.19 [30142.16; 39854.66]	0.843
	R2 (Liconsa)	34319.38 [30358.04; 37704.27]	37770.12 [30828.63; 40329.28]	0.308
	T (Lafepe)	36671.12 [32369.49; 39555.41]	35418.02 [30685.43; 38967.35]	0.799
%AUCextrap (%)	R1 (Elea)	1.32 [1.19; 1.88]	1.81 [1.44; 2.86]	0.010***
	R2 (Liconsa)	1.29 [0.87; 1.65]	1.67 [1.36; 2.06]	0.018***
	T (Lafepe)	1.31 [0.89; 1.89]	1.64 [1.47; 2.61]	0.022***
λ (1/h)	R1 (Elea)	0.0590 [0.0542; 0.0666]	0.0557 [0.0473; 0.0595]	0.070
	R2 (Liconsa)	0.0633 [0.0563; 0.0685]	0.0551 [0.0477; 0.0632]	0.022***
	T (Lafepe)	0.0621 [0.0540; 0.0673]	0.0546 [0.0480; 0.0640]	0.113
t1/2 (h)	R1 (Elea)	11.75 [10.41; 12.79]	12.45 [11.65; 14.67]	0.070
	R2 (Liconsa)	10.94 [10.12; 12.32]	12.59 [10.97; 14.54]	0.023***
	T (Lafepe)	11.15 [10.29; 12.83]	12.69 [10.83; 14.45]	0.113
CL/F (L/h)	R1 (Elea)	1.88 [1.76; 1.98]	2.26 [1.83; 2.79]	0.025***
	R2 (Liconsa)	1.90 [1.78; 2.16]	2.15 [1.93; 2.64]	0.095
	T (Lafepe)	1.81 [1.72; 2.19]	2.30 [1.78; 2.73]	0.039***
CL/F II (L/h/kg)	R1 (Elea)	0.0280 [0.0261; 0.0315]	0.0293 [0.0251; 0.0332]	0.843
	R2 (Liconsa)	0.0291 [0.0265; 0.0329]	0.0265 [0.0248; 0.0324]	0.314
	T (Lafepe)	0.0273 [0.0253; 0.0309]	0.0282 [0.0257; 0.0326]	0.799
Vz/F (L)	R1 (Elea)	33.38 [27.35; 34.47]	43.09 [35.89; 48.08]	< 0.001***
	R2 (Liconsa)	30.54 [27.32; 36.35]	41.51 [38.10; 44.68]	< 0.001***
	T (Lafepe)	31.29 [25.57; 37.63]	39.33 [35.66; 44.07]	0.003***
Vz/F II (L/kg)	R1 (Elea)	0.48 [0.41; 0.54]	0.51 [0.49; 0.58]	0.062
	R2 (Liconsa)	0.47 [0.42; 0.53]	0.49 [0.48; 0.55]	0.067
	T (Lafepe)	0.47 [0.41; 0.50]	0.51 [0.43; 0.61]	0.129
Dose (mg/kg)#	R1 (Elea)	1.45 [1.40; 1.62]	1.21 [1.12; 1.47]	0.003***
	R2 (Liconsa)	1.48 [1.41; 1.60]	1.20 [1.12; 1.46]	0.002***
	T (Lafepe)	1.49 [1.41; 1.61]	1.20 [1.12; 1.47]	0.002***
Weight (kg)#	R1 (Elea)	69.05 [61.88; 71.25]	82.90 [67.85; 89.75]	0.003***
	R2 (Liconsa)	67.90 [62.38; 70.98]	83.40 [68.35; 89.35]	0.002***
	T (Lafepe)	67.35 [62.28; 71.00]	83.10 [68.20; 89.75]	0.003***

* Expressed as median [Q1; Q3];

** Mann-Whitney Rank Sum Test;

*** p < 0.05.

# For dose normalisation weight was measured at each study period, which explains the variation in weight and dose (mg/kg) parameters between formulations.

tmax: time to reach Cmax; Cmax: maximum concentration; Cmax/D: maximum concentration corrected by dose according to weight; AUC84h: area under the curve up to the last measurable concentration; AUC84h/D: area under the curve up to the last measurable concentration corrected by dose according to weight; AUC∞/D: area under the curve up to infinity; AUC∞: area under the curve up to infinity corrected by dose according to weight; %AUCextrap: extrapolated percentage of AUC∞ in relation to AUC84h; λ: apparent elimination constant; t1/2: elimination half-life; CL/F: apparent total body clearance; CL/F II: apparent total body clearance as a function of weight; Vz/F: apparent volume of distribution; Vz/F II: apparent volume of distribution as a function of weight.

Differences in tmax between sexes were observed for the Elea and Liconsa formulations, suggesting a slower absorption rate in women. The t1/2 was lower in women for the Liconsa formulation.

Despite its influence on pharmacokinetic absorption parameters (Cmax and AUC) and apparent difference in impact between the drug formulations studied, sex did not significantly affect the bioequivalence analysis. The linear mixed model for the AUC84h showed no significant sex effect (p = 0.1030) or sex-by-formulation interaction (p = 0.4041). For Cmax, a statistically significant sex effect was observed (p = 0.0058), but the interaction with formulation was not significant (p = 0.4690). The 90% CI for the ratio of geometric mean ratios remained within the regulatory acceptance (80-125%) for all comparisons across sex groups: men [T vs. R1 (103.42; 115.82); T vs. R2 (94.20; 105.49); R2 vs. R1 (103.75; 116.19)]; women [T vs. R1 (99.47; 110.84); T vs. R2 (97.11; 108.21); R2 vs. R1 (97.03; 108.13)].

*Security analysis* - During the study, a total of 33 adverse events were observed. No serious adverse event was observed, and all reported adverse events were classified as mild (94%) or moderate (6%). Of these events, 21 (64%) were evaluated as having some degree of causality related to the use of BZN. Among them, 11 events (52%) were deemed to have probable or possible causality, consisting of ten cases of headache and one case of vomiting. In a small number of cases, headache was managed with a single oral dose of dipyrone (1 g). No other concomitant medications were required for the management of adverse events. The remaining 10 events (48%) were classified as having conditional causality, which included one case of diarrhoea (3%) and laboratory findings of altered liver enzymes following the treatment periods. There were four events (12%) of elevated gamma-glutamyl transferase (GGT), three (9%) of elevated alanine transaminase (ALT), and two (6%) of elevated aspartate transaminase (AST). These transient increases in liver enzymes were classified as a conditional adverse event due to the absence of clinical symptoms and the isolated nature of the laboratory findings.

Regarding formulation-specific causality, among the 11 adverse events with possible or probable causality: 55% occurred following the administration of formulation R2 (Liconsa), 36% after formulation T (Lafepe), and 9% after formulation R1 (Elea). All adverse events resolved completely, and no long-term effects were observed.

In terms of sex distribution, 60% of the adverse events classified as probable, possible, or conditional causality occurred in women. However, the overall incidence of adverse events between sexes was not statistically significant [relative risk (RR) = 1.23; 95% CI: 0.54-2.80].

## DISCUSSION

To our knowledge, this is the first study to compare three currently available BZN formulations, integrating pharmacokinetic evaluation with formal assessments of pharmaceutical equivalence and bioequivalence in healthy participants. For the past 40 years, pharmacokinetic data on BZN has relied on formulations previously produced by Roche, which are no longer available. The most recent formulations, produced by Lafepe (Brazil), Elea (Argentina) and Liconsa (Spain), have not been evaluated for bioequivalence. The lack of published information regarding the interchangeability of these formulations hinders an adequate comparison of results obtained from studies that used different formulations.

*In vitro* tests demonstrated that the three drug formulations showed no significant differences in physicochemical tests and were considered pharmaceutical equivalents. However, dissolution profile analysis showed that the Lafepe and Elea formulations presented equivalent dissolution behaviour, whereas the Liconsa formulation exhibited a different profile, characterised by a faster BZN release. The Liconsa formulation achieved more than 80% drug release within 15 min, compared with approximately 30 min for Elea and 60 min for Lafepe. After 60 min, the unreleased fraction was close to 10% for all three formulations.

Despite the difference observed in the dissolution test, the products were found to be bioequivalent *in vivo*. The ratios for absorption parameters Cmax and ASC were close to 1 for all comparisons between formulations (T/R1; T/R2 and R2/R1), and the 90% CI for the geometric mean ratios fell within the regulatory accepted range of twenty percent variation: 0.80 to 1.25.^20^ ^23^ ^24^

The observed discrepancy between the in vitro (dissolution) and *in vivo* (bioequivalence) results is not entirely clear. However, two hypotheses may explain this difference. The first relates to the methodology used in the dissolution test. As already mentioned, a pharmacopeial method has not been established for assessing pharmaceutical equivalence in BZN, and the method used in this study may not have sufficient discriminatory power for these formulations. Furthermore, the exact composition and manufacturing processes of the formulations are not publicly available, which may influence dissolution behaviour. Further evaluation of this hypothesis is warranted.

The second hypothesis, which we consider more relevant, concerns the administration method used during the clinical trial. All three BZN formulations were administered within 30 min after a high-fat, high-calorie meal (872.4 calories, with 53.0% fat). The presence of food can delay gastric emptying, postponing the drug's arrival at the intestinal absorption site.^26^ ^27^ ^28^ According to FDA data, administering a 100 mg BZN tablet with a high-fat, high-calorie meal (approximately 1034 total kcal, 67 kcal from fat, 42 kcal from carbohydrates, 59 kcal from protein) does not alter Cmax or AUC, but delays tmax by approximately 80 min.^29^ Similarly, in a study comparing Lafepe BZN pharmacokinetics under feed and fasting conditions, tmax was delayed by 90 min with a meal (fed = 5.00 h and fasted = 3.5 h).^15^

The dissolution test simulated the gastric environment, including pH and motility. Within 60 min, 90% or more of the BZN had already been released from all three formulations. Therefore, the delay in drug absorption due to food intake may have masked differences seen in the dissolution test, acting as a rate-limiting step, and equalising the absorption profiles. This finding has important clinical implications. Although food does not significantly affect BZN bioavailability, the drug is typically administered after meals to minimise potential gastric adverse effects (as recommended in the Elea and Lafepe leaflets). As a result, despite differing dissolution rates, the absorption of BZN in the presence of food is likely to be similar across the three formulations in clinical settings.

The pharmacokinetic parameters observed in this trial were similar to those reported in previous studies, after single dose, for bioavailability and elimination parameters,^17^ ^30^ ^31^ even those using formulations no longer available on the market.^32^ For tmax, we observed higher values, which may be explained by the administration of BZN formulations after a high-fat meal. Silveira et al. also suggested the possibility of a higher absorption rate in patients.^17^ Additionally, Wiens et al. also found limited variability in BZN pharmacokinetics across published studies between 1979 and 2016.^30^

Recently, the bioavailability of two BZN formulations (Lafepe vs. Elea) were compared in 13 adults with chronic *T. cruzi* infection (nine women and four men), after a single 100 mg oral dose under fasting conditions.^16^ In agreement with our findings, they reported similar Cmax and tmax values between formulations, although the mean values differed. The tmax was 42% lower in the Hernández study, likely due to fasting administration. Cmax was 28% higher in patients, suggesting potentially increased absorption in this population, a hypothesis requiring further investigation. Supporting this, a higher absorption constant (Ka) was observed in chronically *T. cruzi*-infected Swiss mice compared to healthy controls.^33^

In contrast to our results, Hernández et al. reported greater variability in pharmacokinetic parameters (Cmax 19.9% vs. 9.44%; AUC 40.7% vs. 6.32%), and shorter t1/2 values (8.0 h for Elea and 9.1 h for Lafepe), as well as lower AUC values (32.0 and 37.5 h*ng/mL, respectively for Elea and Lafepe).^16^ Differences in blood sampling schedules may account for these differences. In our study, the elimination phase of BZN was best characterised after 24 h in 70% of participants, and after 36 h in 60%. The lack of more samples after 24 h in the Hernández's study (only one sample was obtained) may have led to inaccurate estimates of the elimination phase, using data still in the distribution phase, which would have generated a high λ and a t1/2 below that commonly found. We observed in our study that only about 24% of the AUC was calculated based on the first 8 h, suggesting that a collection window with fewer points in the distribution and elimination phases (in the Hernández study only three points were obtained after 8 h versus nine points in our study) could have underestimated the AUC and increased the observed variability. However, the possibility of higher variability in patients cannot be ruled out and, as highlighted by Hernández et al., there is still a lack of information regarding factors contributing intra-individual to variability, as well as the influence of population characteristics such as sex, age, and disease status.

Pharmacokinetic data in patients with chronic CD treated with the Lafepe formulation were reported.^17^ Although this study was conducted in infected patients under treatment conditions, the exposure parameters observed were broadly consistent with those obtained in the present study following single-dose administration. In agreement with our findings, a variation between sexes was also observed for the pharmacokinetic absorption parameters after administration of fixed doses of BZN.

It is well established that demographic factors like sex can significantly impact drug absorption, distribution and elimination.^34^ ^35^ In a study involving eight healthy participants (four women and four men), Molina et al. found that BZN Cmax was 81% higher in women (p = 0.02), and that Vz/F was 42% higher in men (p = 0.02) after a single 100 mg dose of Elea BZN.^31^

Our study also assessed the impact of sex on BZN pharmacokinetics for the three formulations. In agreement with Molina and Silveira,^17^ ^31^ we found that BZN plasma concentrations were higher in healthy women. The higher plasma exposure observed in women may also be partially explained by differences in body weight, as lower body weight may lead to relatively higher drug exposure when dosing is not adjusted by weight. The Vz/F were significantly higher in men for all formulations (between 26 and 36%). Cmax were significantly greater in women for all formulations, but AUC were significantly greater in women for Elea and Lafepe formulations.

Interestingly, the influence of sex varied between formulations. Significant sex-related differences in tmax were observed for Elea and Liconsa formulations, but not for Lafepe, suggesting a slower absorption rate in women for these formulations. Women would have a slower gastric emptying time compared to men,^36^ which agrees with what was observed for the Elea and Liconsa formulations. Additionally, only Elea and Lafepe formulations exhibited significant sex differences for AUC and clearance (CL/F; possibly influenced by AUC), whereas only Liconsa formulation showed significant differences in elimination variables λ and t1/2. These observations could be attributed to aleatory variability of the data or to reflect a true sex-by-formulation effect.

The potential impact of the sex-by-formulation effects on bioequivalence studies has been discussed by some authors.^37^ ^38^ ^39^ In agreement, regulatory agencies such as Anvisa and FDA recommend that bioequivalence studies included balanced male and female participation, unless the drug is intended solely for one sex, such as for hormonal contraceptives. In our study, no significant sex-by-formulation interactions were found, reinforcing the interchangeability of the three formulations across both sexes.

Regarding adverse events, 60% of those classified as probable, possible or conditional causality were observed in women. Similarly, other authors were reported a higher incidence of adverse events and treatment discontinuation among women in a cohort of 195 CD patients treated with BZN.^40^ The reason for this sex difference is still unclear. Consistent with the higher plasma exposure observed in women in the present study, a recent clinical pharmacokinetic analysis in patients with chronic CD treated with the Lafepe formulation also reported higher BZN concentrations in women, largely explained by lower body weight.^17^ The study further showed that higher plasma exposure, regardless of sex, was associated with an increased risk of skin reactions.^17^ These findings suggest that weight-based dosing adjustments, rather than sex-specific dosing, may be more relevant for optimising treatment safety. Although differences in the frequency of adverse events were observed among formulations, these findings should be interpreted with caution due to the small sample size and the exploratory nature of the safety analysis. The study was not powered to detect statistically significant differences in safety outcomes between formulations.

Importantly, the present study focused on the 100 mg BZN formulation, which is commonly used in adult treatment. Further studies evaluating the pharmaceutical characteristics and bioequivalence of the 12.5 mg formulation used in paediatric populations would be valuable.

*In conclusion* - This was the first study to demonstrate bioequivalence among the three 100 mg BZN formulations currently available on the global market. This finding holds both clinical and economic relevance, as it supports a greater distribution of all three drugs across different markets, improving patient access. It also has scientific relevance, allowing for the comparison of results from different clinical trials, even when conducted with different formulations. Additionally, the study highlighted that sex may be a relevant factor contributing to BZN pharmacokinetic variability, which could have clinical implications, since women would be subjected to greater exposure to the drug in fixed-dose regimens.

## Data Availability

The data that support the findings of this study are not openly available due to reasons of sensitivity and are available from the corresponding author upon reasonable request. Data are located in controlled access data storage at Fundação Oswaldo Cruz.
