# Supplementary material for: Comparative bioavailability of three benznidazole formulations in healthy individuals: a randomised study
Source: Mem Inst Oswaldo Cruz. 2026 Jul 10;121(Suppl 2):e250307. doi: 10.1590/0074-02760250307 (PMC13361134; doi:10.1590/0074-02760250307)
Supplement: Peer review history

# Reviewer report: FIRST REVIEW ROUND - EDITOR AND REVIEWERS COMMENTS

## EDITOR COMMENTS

Abstract: "The BZN Liconsa demonstrated faster in vitro dissolution". This is not described in the Methods of the Abstract.

Abstract. "Plasma concentrations of BZN were higher in women". For all formulations? How much higher? Include data.

Introduction "burdens," Should be a full stop and not comma.

Introduction "over 75 million people are at risk" The last number is 100 million. (https://www.who.int/news-room/fact-sheets/detail/chagas-disease-(american-trypanosomiasis))

Introduction "It acts by suppressing circulating forms of *T. cruzi*, potentially reducing or eliminating parasitemia". Does it mean that BZN has no activity against the intracellular (amastigote) form?

Introduction "except in advanced chronic Chagas heart disease, where effectiveness tends to diminish with longer infection duration". The BENEFIT trial demonstrated that BZN was effective to decrease parasitemia. BZN failed to show benefit on the studied clinical endpoints. It would be better to clarify this point.

Introduction "For over four decades, PK data were based on formulations that are no longer available, such as Rochagan® and Radanil®, previously produced by Hoffmann-La Roche". There are some papers already published with PK data for LAFEPE and ELEA formulations (example: Silveira GPE. J Antimicrob Chemother. 2025 Nov 24: dkaf416) Please, discuss.

Methods: "T1/2" should read "t1/2"

Methods: Brazil is not signatory of the Declaration of Helsinki and the last version is 2024.

Methods: "The study protocol was published in the Brazilian Registry of Clinical Trials (ReBEC) with the following ID: 63382222.4.0000.80981". Please include the link.

Results: "This difference can be attributed to the average body weight of male participants, which was approximately 19% higher than that of females. Consequently, the weight-adjusted dose (mg/kg) was about 18% lower in men". As the beginning of the paragraph sates higher concentrations in women, it would be better to describe a lower weight in women and consequent larger weight-adjusted dose in women.

Results: There was a difference in Tmax between sexes that was not discussed.

Discussion. First paragraph. There are already some publications about PK using the current available BZ formulations.

Discussion. "The pharmacokinetic parameters observed in this trial were similar to those reported in previous studies [24,25], even those using formulations no longer on the market [26]." Please, rephrase this sentence to be clear which PK parameters are similar to studies that used the same formulation studied in the present paper, and the ones similar to papers that used other formulations.

Discussion. Please include a discussion comparing your results to a recent paper which included patients with CD. Silveira GPE. J Antimicrob Chemother. 2025 Nov 24: dkaf416.

Discussion "Although women exhibit higher BZN plasma concentrations following single-dose administration, the correlation between this higher exposure and adverse events remains uncertain [35]. Larger clinical studies in patients undergoing full treatment could help determine whether these sex-based differences impact therapeutic efficacy and safety at steady-state concentrations." In fact, a clinical study was just published and should be included in this discussion. Silveira GPE. J Antimicrob Chemother. 2025 Nov 24: dkaf416

Author contribution "G.P.E.S., R.E., R.M.S. and M.A.V.S. designed the research." Please clarify who RMS and MAVS stand for among the paper co-authors.

Data availability. Do you plan to include your data in the ARCA repository?

Figure 4. Use different colors for male and female.

Some Figures repeat information already presented by Tables. Please, suppress redundant information.

## REVIEWER #1

1. Abstract

1.1. In the non-compartmental pharmacokinetic analysis, the following parameters are considered: AUC (area under the curve), Cmax, Tmax, elimination half-life (t₁/₂), mean residence time (MRT), and clearance (CL). In this context, it is noted that the abstract does not report the latter parameter, which should be included to provide a more complete summary and to facilitate future research on the topic.

2. Introduction

2.1. Considering the sentence "However, BZN is more frequently used due to its simpler dosing regimen, shorter treatment duration, and potentially better tolerability regarding adverse events", it is noted that differences in dosing regimens between the drugs are mentioned. However, according to the current Brazilian guideline (Clinical Protocol and Therapeutic Guidelines for Chagas Disease, 2018 – Ministry of Health), the study context does not support any distinction in posology or treatment duration between these therapies. The authors are therefore requested to revise this sentence to improve clarity and ensure accurate interpretation by readers.

3. Material and Methods

3.1. Considering the critical role of validated analytical methods and calibrated equipment in bioequivalence studies, the authors are requested to provide calibration records for the equipment used, covering the period immediately preceding and during the study execution, to ensure metrological traceability, data reliability, and the validity of the reported results.

3.2. The authors are requested to provide the package inserts of the three drugs evaluated, as well as detailed information on the batches used in the study and the corresponding monetary value in US dollars at the time the study was conducted, in order to enhance methodological transparency and facilitate verification and interpretation of the data by future readers.

3.3. It is requested that, between lines 151 and 158, the manuscript specify whether any dietary restrictions were applied to the study participants during recruitment and study conduct, including the type of diet implemented and its methodological justification.

3.4. Clarification is requested regarding the rationale for administering the drug "30 minutes after a standardized high-fat meal – line 161", considering that, in clinical practice, individuals undergoing treatment for *Trypanosoma cruzi* infection are generally advised to avoid high-fat foods. In this context, it is essential that the authors explicitly present the methodological justification for this choice and discuss its compatibility with standard clinical management, in light of current care recommendations. Furthermore, this clarification is critical to support the interpretation of the causality of the observed adverse events, allowing distinction between effects attributable to the drug administration and those potentially related to the ingestion of the standardized high-fat meal provided 30 minutes prior to dosing.

3.5. Considering the sentence "Following each BZN administration, participants were clinically observed for 24 hours", clarification is requested regarding how this clinical monitoring was conducted, specifying whether the assessment was performed by a physician and/or nurse, as well as the location where the observation took place. Additionally, it is requested to indicate whether participant hospitalization was required during this period, and if so, to describe the location and the hospitalization protocol applied.

3.6. Regarding the sentence "… laboratory tests after the clinical phase …", it is requested that a detailed table of all laboratory tests performed be provided, including a comparison of participants' plasma parameters before and after drug administration, accompanied by the corresponding medians and interquartile ranges (IQR, 25–75%), stratified by sex.

3.7. Clarification is requested regarding the methodological choice to recruit participants exclusively from a single geographic location in Brazil — "A total of 42 healthy participants residing in Campinas, São Paulo, Brazil were enrolled in the bioequivalence study – line 264" — since such a strategy may introduce selection bias and consequently limit the generalizability of the findings to other populations and regional contexts.

3.8. The authors are requested to present the subgroup consisting of 22 female participants, including the medians and interquartile ranges (IQR, 25–75%) for the variables age and body mass index (BMI), following the description of the study participants (line 267).

3.9. It is requested to describe how the dose was adjusted to participants' body weight (mg/kg), including the criteria used for dose calculation and its application throughout the study.

3.10. It is requested that, between lines 316 and 318, the manuscript specify whether the use of concomitant drugs was necessary to manage the adverse events observed in the study population, or, alternatively, if no additional pharmacological intervention was required.

3.11. It is requested that the manuscript specify whether the use of concomitant drugs was necessary to manage the adverse events observed in the study population, or, alternatively, if no additional pharmacological intervention was required.

3.12. It is noted that, between lines 329 and 359, no bibliographic references were provided to support some of the points discussed in the study. Although the literature on this topic is limited, a simple search in PubMed identified 63 articles related to the subject, which underscores the need to include relevant references to adequately substantiate the discussion presented.

3.13. It is requested that, on lines 364 and 365, where the expression "According to FDA data …" appears, the corresponding bibliographic reference be explicitly provided, in order to ensure proper traceability and support for the cited information.

## REVIEWER #2

1. General Considerations

The revised manuscript is of significant scientific interest, presenting the results of a study comparing the bioavailability of three marketed 100 mg benznidazole formulations.

2. Abstract Adequacy

The abstract is concise and well-structured. However, in the Objective section (line 28), it should be explicitly stated that the bioequivalence assessment refers only to the 100 mg formulations, not to all available formulations. Similarly, in the Main Conclusion (line 42), clarify that the findings pertain specifically to the 100 mg benznidazole formulation.

3. Introduction

Lines 59–62: It is strongly recommended to incorporate the latest epidemiological data published by PAHO/WHO (Update on Chagas Disease Estimates in Endemic Countries of the Americas, 2018. OPS/CDE/VT/25-0005 – Pan American Health Organization, 2025).

Lines 67–68: When mentioning the higher frequency of benznidazole use compared to nifurtimox, emphasize that this is primarily due to its greater market availability. The PAHO/WHO 2018 guidelines recommend both benznidazole and nifurtimox interchangeably as first-line treatment options.

4. Results

For better visualization, include a flowchart illustrating sample selection in the Bioequivalence Analysis section (line 263).

Safety Analysis: Why was the increase in liver enzymes classified as a "conditional adverse event," given that this is a well-documented clinical adverse effect (lines 316–318)? Were baseline laboratory tests performed on volunteers? This should be clarified, as hepatic and gastrointestinal diseases are listed among exclusion criteria (Study Population, Blood Sampling, and Adverse Event Monitoring, line 153).

Regarding the occurrence of adverse events differing among formulations (lines 308–322), an explanation is warranted. Although the sample size (N=11) is small, the differences (55%, 36%, and 9% for Liconsa, Lafepe, and Elea, respectively) merit discussion. Was any statistical analysis performed to assess these differences?

5. Discussion

Line 341–342: The in vitro dissolution differences are not only between Lafepe and Liconsa but also between Lafepe–Elea and Liconsa.

From line 424 onward, where sex-related variability is discussed, no mention is made of weight, despite its inclusion in the results.

Among study limitations, note that only the 100 mg formulation was evaluated; the 12.5 mg formulation remains to be analyzed.

6. Conclusions

Line 451 should clearly state that the conclusions refer to the 100 mg benznidazole formulations.

7. Figures

There are two identical Figure 1s on pages 38 and 43. The title refers to dissolution profiles of the three formulations; it should specify that this is a pharmaceutical equivalence test.

Additionally, consider including a figure comparing the pharmaceutical equivalence test and the bioequivalence analysis in terms of dissolution profiles.

## AUTHORS' RESPONSE TO THE REVIEWERS

**Dear Editor and Reviewers,**

We would like to sincerely thank you for the time and effort dedicated to reviewing our manuscript, as well as for your valuable comments and suggestions. We believe that your contributions have significantly improved the clarity, scientific rigor, and overall quality of the manuscript.

All comments were carefully considered, and the manuscript was revised accordingly. Detailed responses to each point raised by the reviewers are provided below, indicating how the manuscript was modified or, when applicable, explaining the rationale for maintaining the original approach.

We hope that the revised version adequately addresses all concerns raised and meets the standards of the journal.

Thank you once again for your thoughtful and insightful review.

Sincerely,

The Authors.

**I. Editor comments**

**I.1.** Abstract: "The BZN Liconsa demonstrated faster in vitro dissolution". This is not described in the Methods of the Abstract.

We thank the reviewer for this comment. A sentence indicating that in vitro dissolution testing was performed as part of the pharmaceutical equivalence assessment has been added to the Methods section of the Abstract.

**I.2.** Abstract. "Plasma concentrations of BZN were higher in women". For all formulations? How much higher? Include data.

We thank the reviewer for this important observation. The statement in the Abstract was revised to clarify that sex-related differences were observed for the Elea and Lafepe formulations, while no statistically significant difference was observed for Liconsa.

**I.3.** Introduction "burdens," Should be a full stop and not comma.

We thank the reviewer for identifying this typographical error. The punctuation was corrected.

**I.4.** Introduction "over 75 million people are at risk" The last number is 100 million.

We thank the reviewer for pointing this out. The estimate was updated according to the most recent PAHO/WHO documents.

**I.5.** Introduction "It acts by suppressing circulating forms of *T. cruzi*, potentially reducing or eliminating parasitemia". Does it mean that BZN has no activity against the intracellular (amastigote) form?

We thank the reviewer for this important comment. We agree that the original wording could be misinterpreted as suggesting that benznidazole acts only on circulating trypomastigotes. The U.S. Food and Drug Administration (FDA) label for benznidazole explicitly states that the drug is active against all three stages of *T. cruzi* (trypomastigotes, amastigotes, and epimastigotes) though sensitivity may vary by strain. However, the presence of dormant, non-replicative amastigotes can limit the effectiveness of therapy and contribute to persistence and relapse. The sentence in the Introduction has been revised to clarify this point and avoid potential misinterpretation.

**I.6.** Introduction "except in advanced chronic Chagas heart disease, where effectiveness tends to diminish with longer infection duration". The BENEFIT trial demonstrated that BZN was effective to decrease parasitemia. BZN failed to show benefit on the studied clinical endpoints. It would be better to clarify this point.

We agree with the reviewer and have clarified the interpretation of the BENEFIT trial.

**I.7.** Introduction "For over four decades, PK data were based on formulations that are no longer available, such as Rochagan® and Radanil®, previously produced by Hoffmann-La Roche". There are some papers already published with PK data for LAFEPE and ELEA formulations (example: Silveira GPE. J Antimicrob Chemother. 2025 Nov 24:dkaf416) Please, discuss.

We thank the reviewer for this comment. The manuscript was submitted before the publication of Silveira et al. (2025), which reports pharmacokinetic data without an explicit bioequivalence assessment, and has now been included. Historically, most PK data were generated using Roche formulations that are no longer available, while more recent studies evaluated Lafepe alone or compared Lafepe and Elea. To our knowledge, no published studies have directly compared Lafepe and Liconsa formulations. The present study provides the first comparative PK and bioequivalence assessment of three currently available formulations (Lafepe, Elea/Abarax, and Liconsa) in healthy volunteers. The manuscript has been revised accordingly.

**I.8.** Methods: "T1/2" should read "t1/2"

The notation was standardized throughout the manuscript to t1/2. "Tmax" was also changed to "tmax".

**I.9.** Methods: Brazil is not signatory of the Declaration of Helsinki and the last version is 2024.

We thank the reviewer for this clarification. The text was revised accordingly.

**I.10.** Methods: "The study protocol was published in the Brazilian Registry of Clinical Trials (ReBEC) with the following ID: 63382222.4.0000.80981". Please include the link.

The requested link was added.

**I.11.** Results: "This difference can be attributed to the average body weight of male participants, which was approximately 19% higher than that of females. Consequently, the weight-adjusted dose (mg/kg) was about 18% lower in men". As the beginning of the paragraph sates higher concentrations in women, it would be better to describe a lower weight in women and consequent larger weight-adjusted dose in women.

We agree that the paragraph could be clearer and revised the wording.

**I.12.** Results: There was a difference in Tmax between sexes that was not discussed.

We thank the reviewer for noting this point. The results section was expanded to address the observed sex-related differences in tmax.

**I.13.** Discussion. First paragraph. There are already some publications about PK using the current available BZ formulations.

We thank the reviewer for this comment. The manuscript was submitted before the publication of Silveira et al. (2025), which has now been included in the revised version. We would like to clarify that the original statement referred specifically to the fact that this is the first study comparing three currently available benznidazole formulations and combining a full pharmacokinetic and bioequivalence evaluation in healthy volunteers. The sentence has been slightly rephrased to improve clarity.

**I.14.** Discussion. "The pharmacokinetic parameters observed in this trial were similar to those reported in previous studies [24,25], even those using formulations no longer on the market [26]." Please, rephrase this sentence to be clear which PK parameters are similar to studies that used the same formulation studied in the present paper, and the ones similar to papers that used other formulations.

There are no clinically meaningful differences between the pharmacokinetic parameters reported in previous studies of Roche benznidazole formulations and those reported in the recent studies by Silveira GPE et al. (The Journal of Antimicrobial Chemotherapy, 2025) and Hernández Y et al. (Memorias Do Instituto Oswaldo Cruz, 2024). All studies consistently report similar values for maximum plasma concentration (Cmax), time to maximum concentration (tmax), and area under the curve (AUC) following a single 100 mg oral dose in adults. We have added the most recent references to the text.

**I.15.** Discussion. Please include a discussion comparing your results to a recent paper which included patients with CD. Silveira GPE. J Antimicrob Chemother. 2025 Nov 24: dkaf416.

The manuscript was submitted before the publication of Silveira et al. (2025). These data have now been incorporated into the Discussion to compare the pharmacokinetic findings for the Lafepe formulation.

**I.16.** Discussion "Although women exhibit higher BZN plasma concentrations following single-dose administration, the correlation between this higher exposure and adverse events remains uncertain [35]. Larger clinical studies in patients undergoing full treatment could help determine whether these sex-based differences impact therapeutic efficacy and safety at steady-state concentrations." In fact, a clinical study was just published and should be included in this discussion. Silveira GPE. J Antimicrob Chemother. 2025 Nov 24: dkaf416

The manuscript was submitted before the publication of Silveira et al. (2025). The discussion was updated to include the recently published clinical study.

**I.17.** Author contribution "G.P.E.S., R.E., R.M.S. and M.A.V.S. designed the research." Please clarify who RMS and MAVS stand for among the paper co-authors.

The data were erroneously inserted and were removed.

**I.18.** Data availability. Do you plan to include your data in the ARCA repository?

We thank the reviewer for the suggestion. At this stage, due to controlled access to individual pharmacokinetic data, the dataset will remain available upon reasonable request to the corresponding author. However, the possibility of deposition in the ARCA repository will be evaluated.

**I.19.** Figure 4. Use different colors for male and female.

Figure 4 was revised to use different colors to distinguish better male and female participants.

**I.20.** Some Figures repeat information already presented by Tables. Please, suppress redundant information.

Figure 5 and Table 6 were removed. At the request of the other reviewers, a new table and a new figure were inserted.

**II. Reviewer 1**

**II.1.** Abstract: In the non-compartmental pharmacokinetic analysis, the following parameters are considered: AUC (area under the curve), Cmax, Tmax, elimination half-life (t1/2), mean residence time (MRT), and clearance (CL). In this context, it is noted that the abstract does not report the latter parameter, which should be included to provide a more complete summary and to facilitate future research on the topic.

We thank the reviewer for this suggestion. Although clearance (CL/F) was calculated as part of the non-compartmental analysis, it was not originally included in the abstract to maintain conciseness and focus on the primary bioequivalence parameters (Cmax, tmax and AUC). Given the 200-word limit for the abstract, only the most relevant results are presented here; however, all data are reported and discussed in the main text and tables 3 and 5.

**II.2.** Introduction: Considering the sentence "However, BZN is more frequently used due to its simpler dosing regimen, shorter treatment duration, and potentially better tolerability regarding adverse events", it is noted that differences in dosing regimens between the drugs are mentioned. However, according to the current Brazilian guideline (Clinical Protocol and Therapeutic Guidelines for Chagas Disease, 2018 – Ministry of Health), the study context does not support any distinction in posology or treatment duration between these therapies. The authors are therefore requested to revise this sentence to improve clarity and ensure accurate interpretation by readers.

We thank the reviewer for this important comment. We agree that, according to the Brazilian Clinical Protocol and Therapeutic Guidelines for Chagas Disease (Ministry of Health, 2018), both benznidazole and nifurtimox are recommended for a treatment duration of approximately 60 days. Therefore, the reference to a shorter treatment duration in the original sentence could lead to misinterpretation and has been removed. The Brazilian guideline indicates that benznidazole may be administered up to three times daily, whereas nifurtimox is recommended in three divided doses per day. In addition, the prescribing information from the manufacturers provides further clarification regarding the usual dosing schedule: benznidazole (Lafepe and Elea formulations) is recommended to be administered in two daily doses, while nifurtimox (Bayer) is recommended to be administered three times daily. Therefore, we will remove the mention of a shorter regimen, but we will retain the observation that the BZN dosage is simpler and better tolerated.

**II.3.** Material and Methods: Considering the critical role of validated analytical methods and calibrated equipment in bioequivalence studies, the authors are requested to provide calibration records for the equipment used, covering the period immediately preceding and during the study execution, to ensure metrological traceability, data reliability, and the validity of the reported results.

We thank the reviewer for this important comment. All analytical procedures used in this study were conducted in a certified analytical and bioanalytical laboratory by Brazilian Health Regulatory Agency (Anvisa), following validated methods and in accordance with Good Laboratory Practice (GLP) principles and applicable Brazilian regulatory requirements. The analytical instruments used during the study were routinely calibrated and maintained according to the laboratory's internal quality assurance system, ensuring metrological traceability and data reliability. Calibration and maintenance records are maintained by the laboratory and are available for audit if required. A clarifying statement has been added to the Methods section.

**II.4.** The authors are requested to provide the package inserts of the three drugs evaluated, as well as detailed information on the batches used in the study and the corresponding monetary value in US dollars at the time the study was conducted, in order to enhance methodological transparency and facilitate verification and interpretation of the data by future readers.

We thank the reviewer for this suggestion. The information regarding the batches used in the study is already included in the manuscript (Methods section). Package inserts are publicly available from the respective manufacturers. Regarding the inclusion of the monetary value of the products, these elements are not typically reported in bioequivalence studies, as they do not affect the pharmacokinetic evaluation of the formulations.

**II.5.** It is requested that, between lines 151 and 158, the manuscript specify whether any dietary restrictions were applied to the study participants during recruitment and study conduct, including the type of diet implemented and its methodological justification.

We thank the reviewer for this comment. Participants were instructed to follow standard dietary restrictions typically applied in pharmacokinetic studies, including overnight fasting prior to drug administration. A clarification has been added to the Methods section.

**II.6.** Clarification is requested regarding the rationale for administering the drug "30 minutes after a standardized high-fat meal – line 161".

We thank the reviewer for this important comment. The administration of the study drug following a standardized high-fat meal was performed according to regulatory recommendations for bioequivalence studies (Brazilian Health Regulatory Agency, Anvisa), which require evaluation under fed conditions to assess potential food effects on drug absorption. Standardized meal conditions are commonly used to reduce variability in drug absorption and to ensure consistency in pharmacokinetic comparisons between formulations. The standardized meal was administered to all participants under the same conditions, minimizing potential confounding effects on the comparison between formulations. The use of a controlled meal in the clinical research setting does not aim to reproduce routine dietary recommendations for patients undergoing treatment for Chagas disease, but rather to ensure standardized experimental conditions during the pharmacokinetic evaluation. A clarifying statement has been added to the Methods section.

**II.7.** Considering the sentence "Following each BZN administration, participants were clinically observed for 24 hours", clarification is requested regarding how this clinical monitoring was conducted.

We thank the reviewer for this comment. The clinical monitoring procedures were already described in the manuscript (lines 171–175). Briefly, participants remained under clinical observation for 24 hours at the clinical research unit, with monitoring performed by trained medical staff, including vital signs assessment, protocol-specified laboratory evaluations, and monitoring for adverse reactions. To improve clarity and facilitate identification of this information, the text has been slightly revised in the Methods section.

**II.8.** Regarding the sentence "… laboratory tests after the clinical phase …", it is requested that a detailed table of all laboratory tests performed be provided.

We thank the reviewer for this suggestion. However, the manuscript already contains a substantial number of figures and tables, and the inclusion of these descriptive data would not add meaningful value to the analysis of the results. The study population consisted exclusively of healthy participants, with only individuals presenting normal test results or no clinically significant alterations being included. A description of the laboratory tests performed was included in the section "Study Population, blood samples and adverse events monitoring".

**II.9.** Clarification is requested regarding the methodological choice to recruit participants exclusively from a single geographic location in Brazil.

Recruitment from a single clinical research center is common practice in bioequivalence studies to ensure standardized study procedures and controlled conditions. This design minimizes variability related to operational factors. A clarification has been added to the Methods section.

**II.10.** The authors are requested to present the subgroup consisting of 22 female participants, including the medians and interquartile ranges (IQR, 25–75%) for the variables age and body mass index (BMI).

We thank the reviewer for this suggestion. Indeed, the study included 22 female participants. As the study was not designed or powered to perform subgroup analyses by sex, a separate table was not considered necessary. However, to provide additional descriptive information, the median age and body weight for the female subgroup have now been included in the Results section. We preferred to include the variable body weight rather than BMI because the medication dose adjustment is based on weight. Women had a lower weight than men, and they were also shorter (median of 1.59m for women (IQR 1.56-1.66); median of 1.77m for men (IQR 1.72-1.81)), which makes the BMI less discriminating between the sexes.

**II.11.** It is requested to describe how the dose was adjusted to participants' body weight (mg/kg).

No dose adjustment was applied for the bioequivalence analysis. The compared formulations were fixed-dose products (100-mg tablets), and each subject received a single dose of each product. For the analysis of sex-related differences, dose normalization by body weight (mg/kg) was performed by dividing the nominal administered dose (100 mg) by each participant's body weight. This normalized dose was then used to adjust the observed pharmacokinetic parameters (e.g., Cmax/normalized dose). An explanation was included in the Methods.

**II.12.** It is requested that, between lines 316 and 318, the percentage values corresponding to each adverse drug event be presented.

We thank the reviewer for this suggestion. As recommended, the adverse drug events have been reformatted to present the values as number and percentage (n, %) immediately following the numerical counts. The corresponding section of the Results (lines 316–318) has been revised accordingly.

**II.13.** It is requested that the manuscript specify whether the use of concomitant drugs was necessary to manage the adverse events observed in the study population.

We thank the reviewer for this comment. In a small number of cases, participants received a single dose of dipyrone (1 g) for the management of headache during the clinical observation period. This information has been added to the manuscript to clarify the management of adverse events.

**II.14.** It is noted that, between lines 329 and 359, no bibliographic references were provided to support some of the points discussed in the study.

We thank the reviewer for highlighting this issue. Additional references have been included in the Discussion to support the statements presented in this section.

**II.15.** It is requested that, on lines 364 and 365, where the expression "According to FDA data …" appears, the corresponding bibliographic reference be explicitly provided.

We thank the reviewer for this observation. The appropriate bibliographic reference supporting the statement based on FDA data has now been included in the manuscript.

**III. Reviewer 2**

**III.1.** Abstract Adequacy: The abstract is concise and well-structured. However, in the Objective section (line 28), it should be explicitly stated that the bioequivalence assessment refers only to the 100 mg formulations, not to all available formulations. Similarly, in the Main Conclusion (line 42), clarify that the findings pertain specifically to the 100 mg benznidazole formulation.

We thank the reviewer for this helpful suggestion. The Objective and the Main Conclusion in the Abstract have been revised to explicitly indicate that the findings refer to the 100 mg formulation.

**III.2.** Introduction. Lines 59–62: It is strongly recommended to incorporate the latest epidemiological data published by PAHO/WHO.

We thank the reviewer for this suggestion. The most recent epidemiological estimates published by PAHO/WHO have now been incorporated.

**III.3.** Introduction. Lines 67–68: When mentioning the higher frequency of benznidazole use compared to nifurtimox, emphasize that this is primarily due to its greater market availability.

We thank the reviewer for this important observation. We agree that both benznidazole and nifurtimox are recommended as first-line treatment options for Chagas disease in PAHO/WHO guidelines and may be used interchangeably in terms of therapeutic efficacy. With regard to availability, benznidazole is currently produced by two manufacturers, whereas nifurtimox is provided by Bayer through a donation program coordinated by the PAHO Strategic Fund, which has expanded access to treatment, particularly for vulnerable populations in endemic regions. In clinical practice, however, the more frequent use of benznidazole has also been associated with its generally simpler dosing regimen and a more favorable tolerability profile compared with nifurtimox, as reported in the literature. To better reflect these aspects, the corresponding sentence in the Introduction has been revised.

**III.4.** Results. For better visualization, include a flowchart illustrating sample selection in the Bioequivalence Analysis section (line 263).

We thank the reviewer for this suggestion. A flowchart illustrating participant enrollment, inclusion in the pharmacokinetic analysis has been added to the manuscript to improve the visualization of the bioequivalence study population (new Figure 2).

**III.5.** Results. Safety Analysis: Why was the increase in liver enzymes classified as a "conditional adverse event," given that this is a well-documented clinical adverse effect (lines 316–318)?

We thank the reviewer for this important comment. Baseline laboratory tests were performed prior to drug administration to ensure eligibility of participants, including evaluation of hepatic parameters. The increase in liver enzymes observed during the study was classified as a conditional adverse event because the clinical relevance could not be definitively attributed to drug exposure based on a single isolated laboratory alteration without clinical symptoms. To clarify this point, the manuscript has been revised to explicitly mention that baseline laboratory tests were performed and to better explain the classification of this event.

**III.6.** Results. Regarding the occurrence of adverse events differing among formulations (lines 308–322), an explanation is warranted.

We thank the reviewer for this observation. The apparent differences in adverse event frequency among formulations should be interpreted with caution due to the small sample size (N=11 per sequence) and the exploratory nature of the safety analysis. No formal statistical comparison was performed for adverse events, as the study was not powered to detect safety differences between formulations. This point has now been clarified in the manuscript.

**III.7.** Discussion. Line 341–342: The in vitro dissolution differences are not only between Lafepe and Liconsa but also between Lafepe–Elea and Liconsa.

We thank the reviewer for this observation. In the in vitro dissolution analysis, the Lafepe and Elea formulations were considered equivalent, whereas the Liconsa formulation showed a different dissolution profile, with a faster benznidazole release. Therefore, the difference was observed between Liconsa and both Lafepe and Elea formulations. We agree that the original wording could lead to ambiguity, and the text has been revised to clarify that Lafepe and Elea showed equivalent dissolution profiles, while Liconsa differed from both formulations.

**III.8.** Discussion. From line 424 onward, where sex-related variability is discussed, no mention is made of weight, despite its inclusion in the results.

We thank the reviewer for this suggestion. The Discussion has been revised to explicitly mention the potential influence of body weight on the observed sex-related variability in benznidazole exposure.

**III.9.** Discussion. Among study limitations, note that only the 100 mg formulation was evaluated; the 12.5 mg formulation remains to be analyzed.

We thank the reviewer for this suggestion. The objective of the present study was specifically to evaluate the pharmaceutical equivalence and bioequivalence of benznidazole 100 mg tablet formulations, which are the strengths most commonly used in adult treatment. Therefore, the evaluation of the 12.5 mg formulation was outside the scope of the present study. However, we agree that the assessment of pediatric formulations represents an important area for future research. To acknowledge this point, a sentence highlighting this knowledge gap has been added to the Discussion section.

**III.10.** Conclusions. Line 451 should clearly state that the conclusions refer to the 100 mg benznidazole formulations.

We thank the reviewer for this suggestion. The Conclusions section has been revised.

**III.11.** Figures. There are two identical Figure 1s on pages 38 and 43. The title refers to dissolution profiles of the three formulations; it should specify that this is a pharmaceutical equivalence test.

We thank the reviewer for this observation. The duplicated figure has been corrected. In addition, the figure title has been revised to clarify that the dissolution profiles correspond to the pharmaceutical equivalence test.

**III.12.** Figures. Additionally, consider including a figure comparing the pharmaceutical equivalence test and the bioequivalence analysis in terms of dissolution profiles.

We appreciate the suggestion; however, the article already presents a substantial amount of data within the proposed objectives. A more detailed comparison of the in vivo data with those observed in vitro may be addressed in a future study.

# Reviewer report: REVIEWERS COMMENTS

## REVIEWER #1

I believe the authors have satisfactorily answered the reviewers' questions and have largely incorporated their suggestions. My position is to accept the manuscript for publication with the changes they made and proceed with the publication process.

## REVIEWER #2

No comments.
